# Determining the quality of life-aged care consumer threshold to identify good quality of life in older adults in long-term care

**DOI:** 10.1007/s11136-026-04280-2

**Published:** 2026-06-06

**Authors:** Jia Song, Julie Ratcliffe, Rachel Milte, Jyoti Khadka

**Affiliations:** 1 Health and Social Care Economics Group, Caring Futures Institute, College of Health and Enablement, Flinders University, Bedford Park, SA Australia Health & Medical Research Building, Sport Road, 5042; 2 Registry of Senior Australians Research Centre, Caring Futures Institute, Flinders University, Adelaide, Australia

**Keywords:** Quality of life, QOL-ACC, Quality indicator, Cut-off value, Aged care, Long term care, ROC curve analysis

## Abstract

**Objective:**

With ageing populations and increasing demand for aged care, quality of life (QoL) has become a central focus of recent policy reforms in Australia and other countries. The Quality of Life-Aged Care Consumer (QOL-ACC), a preference-based measure, was introduced as a mandatory national quality indicator across Australia in 2023 to support assessment and public reporting of variation in QoL across long term aged care facilities (LTCFs). While its national implementation is a significant step forward, further guidance is needed to support the interpretation of QOL-ACC scores in practice. Therefore, this study aimed to identify an empirical reference point on the QOL-ACC to aid interpretation of scores in LTCFs.

**Methods:**

Data were collected through self-reports from LTCFs residents and proxy reports from informal carers. An anchor-based method was used, comparing QOL-ACC summative scores (range: 0 to 24; higher scores indicate better QoL) against two global items of health and QoL. Receiver Operating Characteristic (ROC) curve analysis assessed the discriminative ability of different QOL-ACC scores in identifying residents who rated their global health or quality of life as “good” or better. Sensitivity, specificity, and area under the curve (AUC) were calculated.

**Results:**

Of the total 316 care recipients included (200 [62.5%] self-reports and 116 [37.5%] proxy-reports), the majority were female (64.2%) with a mean age of 84 ± 8.2 years. A QOL-ACC summative score of ≥ 18 was identified as the optimal reference threshold for indicating good quality of life, with a sensitivity of 76%, specificity of 78%, and an AUC of 0.83 (95% CI 0.78—0.87), indicating good discriminative ability.

**Conclusion:**

This study provides a practical and evidence-based interpretive reference point for QOL-ACC scores in long-term care facilities. The identified cut-off may support interpretation of QOL-ACC outcomes in routine quality monitoring and inform care planning, service evaluation, and quality improvement interventions.

## Abstract (plain language summary)

As the population ages, improving quality of life (QoL) in aged care has become a major priority in Australia and other countries. The QOL-ACC is a national quality indicator introduced in 2023 to measure the quality of life of people living in aged care facilities. However, staff and policymakers need clearer guidance on how to understand and use these scores in practice. This study aimed to identify a practical reference point on the QOL-ACC scale. We analysed survey data from 316 long-term care residents and compared QOL-ACC scores with residents’ overall ratings of their health and quality of life. We found that a score of 18 provides a practical reference threshold for interpreting QOL-ACC results. Scores above this level may reflect a good standard of quality of life, while lower scores may indicate poorer QoL. This reference point may help aged care providers and policymakers better understand quality of life outcomes and support improvements in care. This threshold should be used with caution and alongside other quality of life information.

## Introduction

Increasing life expectancy and declining fertility rates have led to rapid population ageing across many Organisations for Economic Co-operation and Development (OECD) countries [1]. Over the last 30 years, the proportion of people aged 65 years and older has risen from 11.7% in 1992 to 18% in 2022 across OECD member countries on average [1]. Japan, Italy, and Greece currently have the highest proportions of older adults, with aged people accounting for between 24% and 29% of their total populations. Australia’s ageing rate is close to the OECD average. As of 2022, one in six Australians (17.1% of the population) were aged 65 years or over, and this figure is projected to rise significantly in the coming decades [2]. The ageing population is expected to drive substantial growth in demand for long-term aged care services, both globally and within Australia [3]. In 2022–2023, more than 1.27 million older Australians accessed aged care services, including long-term residential care, home care, transition care, and home support. Among them, approximately 193,000 people were permanent residents in long-term care facilities (LTCFs), representing about 15% of all aged care users [4]. As the older population continues to grow, the demand for aged care services is expected to grow exponentially.

Aged care is a multi-billion dollar business, requiring substantial financial investment from the government. In Australia, the aged care system is mainly funded through a combination of federal government alongside care recipient co-contributions. In 2022–2023, Australian governments allocated nearly $28.3 billion on aged care, with the largest proportion (58%, or $16.3 billion) spent on long-term residential care [5]. Given the substantial funding from taxpayers in aged care and public interest in quality of services provided in aged care, ensuring that care quality is effectively monitored remains a key policy priority.

In response to recommendations from the Royal Commission into Aged Care Quality and Safety [6], Australian governments have placed a strong focus on improving aged care quality [7]. To ensure better quality of care is provided, the national quality indicator (QI) program was expanded commencing April 2023 to include person-centred outcomes alongside with other nine clinical QIs [8]. Two new mandatory quality indicators developed by our team, the Quality of Life Aged Care Consumers (QOL-ACC) and the Quality of Care Experience Aged Care Consumers (QCE-ACC), were introduced for all residential aged care facilities beginning in April 2023 [9]. The QOL-ACC measures quality of life of older people in terms of six key dimensions: mobility, pain management, emotional wellbeing, independence, social connection, and activities [10]. The QCE-ACC captures aged care consumers’ experiences of aged care services across six attributes: respect and dignity, making own decisions, skill and training of staff, health and wellbeing, social relationships, and lodging complaints in aged care [11]. These indicators emphasise the importance of prioritising the wellbeing and quality of life of older people (aged 65 years and over) as the main objective of Australia’s aged care system [6]. Both instruments provide overall summative quality of life scores as well as a preference-based utility value sets for the purposes of measuring and valuing outcomes within economic evaluation.

This study focuses on the QOL-ACC, a quality of life measure co-designed with older people receiving aged care services. Its validity and reliability have been extensively tested in both home-based and residential care settings [12–16]. While QOL-ACC has strong potential to enhance population-level quality assessment in aged care, its practical application presents challenges when scores are interpreted without clear interpretive guidance. A recent study in residential aged care has highlighted that staff often find QoL scores, including QOL-ACC, difficult to interpret or apply in routine care [17]. To make QOL-ACC more meaningful in routine care, it is essential to determine what constitutes an acceptable care outcome, such as QOL-ACC scores that may be interpreted as indicating good quality of life. This requires establishing a cut-off point above which higher scores may be interpreted as reflecting a good standard of quality of life, and below which lower scores may indicate poorer QoL levels. Establishing such a cut-off does not replace the information provided by the continuous QOL-ACC score. Rather, it provides a simple reference point that can support care staff in identifying lower scores that may indicate potential poor QoL and prompt further assessment or support where needed.

However, a review of existing instruments used to assess quality of life in older populations shows that few have established clear interpretive cut-off points. The most commonly applied generic preference-based instrument in both community and residential aged care is the EuroQol-5 Dimensions (EQ-5D) [18, 19], followed by the Adult Social Care Outcomes Toolkit (ASCOT) [20] and the Health Utilities Index (HUI2/3) [21]. The most widely applied older person–specific instrument across aged care settings is the ICEpop CAPability measure for Older People (ICECAP-O) [22]. While some generic preference-based measures of health-related quality of life, such as the EQ-5D, have proposed cut-offs in specific clinical contexts [23, 24], comparable interpretive reference points have not been established for community or residential aged care–specific quality-of-life instruments. This absence of empirically derived interpretive thresholds represents an important gap in the literature and limits the practical interpretation of aged care–specific QoL instruments.

Further guidance is therefore needed to support the interpretation of QOL-ACC scores in practice. Whilst continuous QOL-ACC scores are appropriate for economic evaluation in the context of quality assessment, an interpretive threshold is important for defining the score range that indicate above which quality of life may be classified as good. In the absence of established quality of life thresholds in residential aged care, this study adopts an exploratory approach to identify a population-specific reference point on the QOL-ACC scale. Accordingly, this study aims to estimate the optimal cut-off point of the QOL-ACC instrument to differentiate between good and poor QOL in residential care settings. As no such threshold currently exists, this represents the first attempt to identify a reference point on a continuous score set for what may be considered ‘good’ QoL amongst older people assessed using the QOL-ACC in aged care. The findings have important implications for aged care administrators and policymakers, who can use the interpretive threshold to support decision-making, improve quality assessment, and inform policies that enhance aged care services and promote high quality aged care.

## Methods

### Surveys, participants, and sample selection

This was a cross-sectional study with 320 older people (65 years and older) living in residential care facilities nationwide in Australia. Data were collected in 2023 from residential aged care facilities and through an online survey for proxy respondents. Older people completed the QOL-ACC either as a self-administered questionnaire or through interviewer administration by trained research staff. Proxy responses were obtained online through a market research panel. Different administration modes may introduce some variation in response patterns; however, their inclusion enhances the real-world relevance and generalisability of the findings.

The study sample was therefore resourced from two survey datasets: self-reported data from older people (*n* = 200) and proxy reported data from informal carers of older people (*n* = 120). Given the high prevalence of dementia among permanent residential aged care residents, not all individuals were able to complete the QOL-ACC independently [25]. In line with national practice, proxy respondents (family members who visit them frequently) were invited to complete the instrument for residents who were unable to self-report, with instructions to rate QoL from the resident’s perspective wherever possible. Inclusion of proxy responses reflects current practice within the National Aged Care Quality Indicator Program, where approximately 20% of QOL-ACC assessments are proxy-completed [8], and supports representation of residents who are unable to self-complete due to cognitive or functional limitations. Accordingly, self- and proxy- reposted data were combined for analysis to reflect the population in which the QOL-ACC is implemented.

**Fig. 1:**
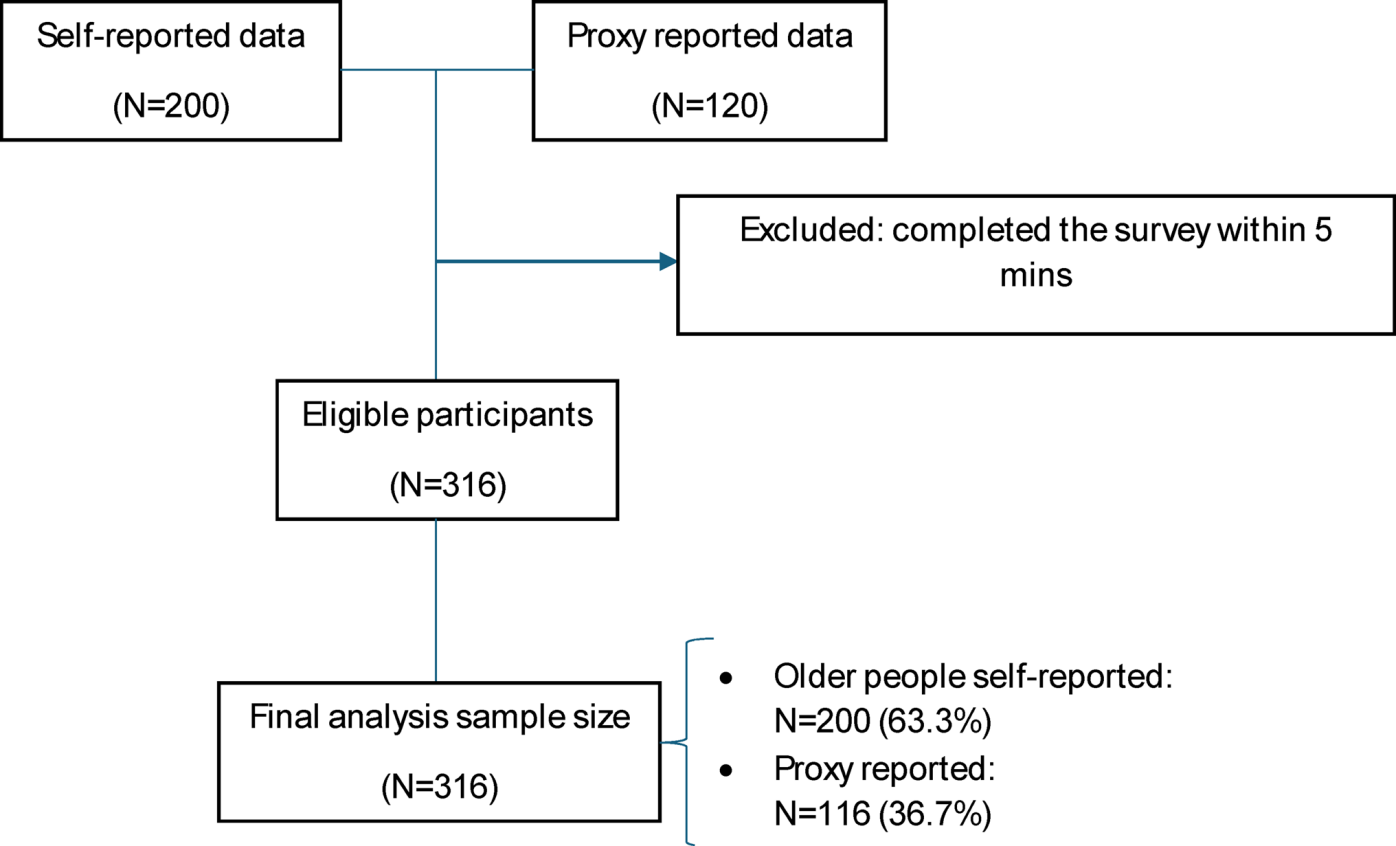
The flowchart of sample selection

We excluded surveys completed in under 5 min (*N* = 4) because this duration was considered insufficient to thoughtfully complete the survey, which included a total of 48 items, with attention and accuracy. The exclusion were all from the proxy group. The mean completion time among included proxy respondents was 28.3 min (median = 14 min, IQR = 9.2–20.0 min). The 5-minute threshold represented the bottom 1% of the completion-time distribution and was applied to identify implausibly rapid responses. This approach is consistent with quality-control practices in online patient-reported outcome (PRO) and quality-of-life research, where respondents typically spend around 16 s per item on average [26]. Our 5-minute threshold for a 48-item questionnaire (6.3s /item) is therefore conservative. The final analysis sample retained 316 participants, of whom 200 (63.3%) were older people self-reported and 116 were proxy reported data (Fig. 1).

### Measures

#### The QOL-ACC instrument

The QOL-ACC measures quality of life of older people across six key dimensions: mobility, pain management, emotional wellbeing, independence, social connection, and activities [10]. Care recipients (or their proxies) are asked to rate their quality of life on a five point response scale reflecting the extent to which each quality of life dimension is achieved (0 = None of the time, 1 = A little of the time, 2 = Some of the time, 3 = Most of the time, 4 = All of the time) describing 15,625 quality of life states in total. The score for each dimension reflects the care recipient’s perceived quality of life in that aspect. The summative score of the QOL-ACC ranges from 0 to 24. A higher QOL-ACC score indicates a better level of quality of life in aged care. The QOL-ACC demonstrated a good internal consistency reliability assessed with Cronbach’s alpha (α = 0.70–0.77) [14, 15], and a strong evidence of construct validity of the QOL-ACC in residential aged care population [14, 16, 27].

#### The global item of quality of life and the global item of health

In addition to the QOL-ACC measurement, data were also collected on care recipients’ rating of quality of life and health using single-item global questions, which are legacy measures that have been previously validated and used extensively in Australian aged care populations [14]. Older people (or their proxies) were asked to rate their overall QoL and health on a five-point scale, ranging from ‘excellent’ to ‘very good,’ ‘good,’ ‘fair,’ and ‘poor.’ Old people’s (or their proxies’) responses to those two global items used as anchors to establish cutoff scores for good quality of life for aged people in residential care because they reflected the disparate values and preference of individuals [28].

### Study design

This cross-sectional observational study was conducted in residential aged care settings, with participants were either older people receiving long-term residential aged care services or carers of the older people in LTCFs. This study aimed to use empirical data to identify a cut-off point above which higher QOL-ACC scores may be interpreted as reflecting good quality of life, and below which lower scores may indicate poorer QoL levels, using global items of QoL and health as the reference points/anchors. To do this, we first categorised responses to the global QoL and health items into four initial groups (Table 1): G1 (good global QoL): rated as excellent, very good or good (*n* = 221); G2 (poor global QoL): rated as fair or poor (*n* = 95); G3 (good global health): rated as excellent, very good, or good (*n* = 181); G4 (poor global health): rated as fair or poor (*n* = 135).

**Table 1:**
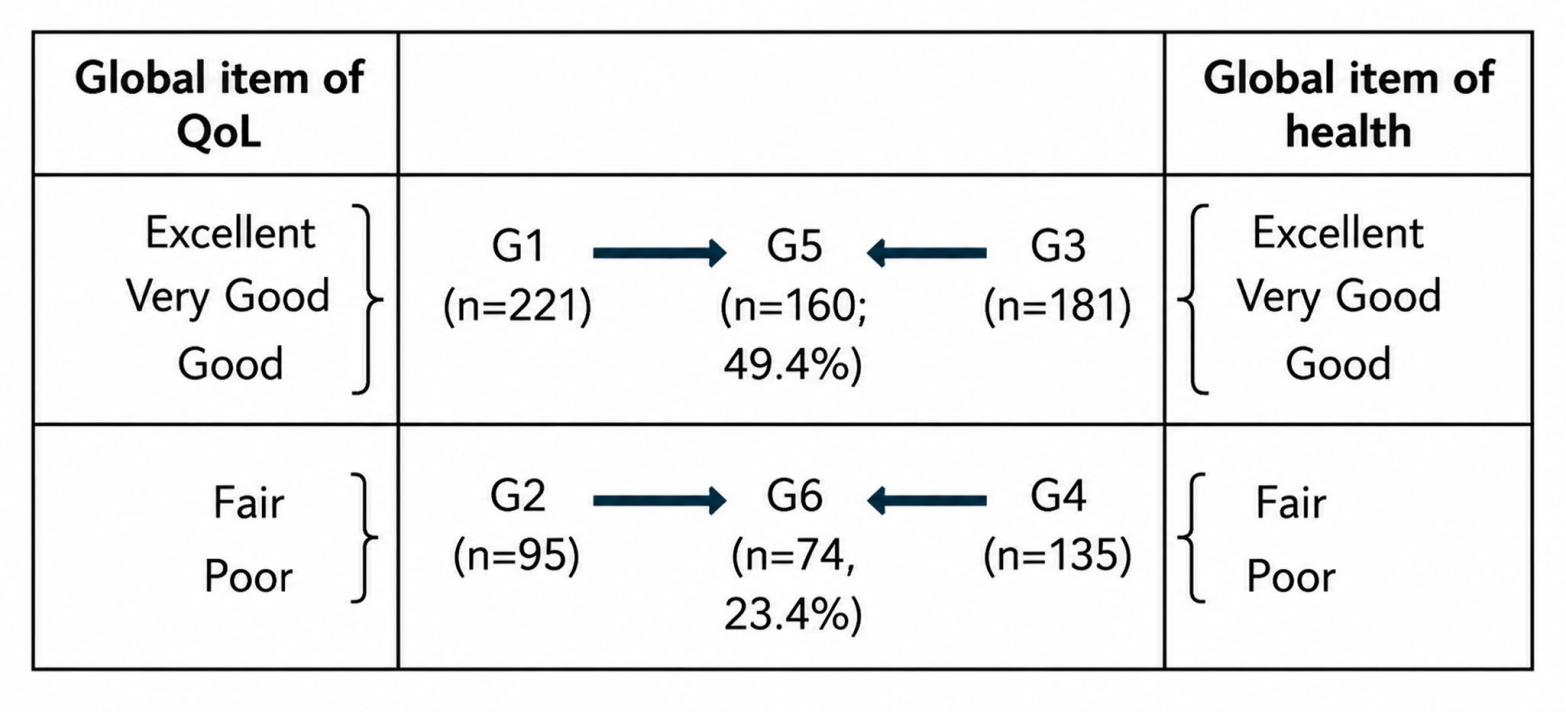
Study sample grouping based on the perceived overall quality of life and health

Based on the definition of good/poor global QoL and health, we then defined two combined groups for ROC analysis (Table 1): G5 (good QoL & health), which was the intersection of G1 and G3 (*n* = 160; 49.4%). As opposed to G5, group G6 (poor QoL & health), which was the intersection of G2 and G4 (*n* = 74; 23.4%). G5 was used as the outcome variable which indicated good QOL outcomes among older people in residential care, while G6 will be used at the outcome variable which indicates poor QOL outcomes.

Among the 221 older people who perceived their general QoL as good or above, 72.4% reported good health (good, very good or excellent), forming group G5 (good QoL & health). The remaining 95 participants perceived their QoL as below good, with a majority (77.9%) reported fair or poor health, and those making up group G6 (poor QoL & health) (Appendix Table 6).

### Receiver operative curve (ROC) analysis

A ROC analysis was conducted to identify the optimal cut-off score for good QOL-ACC outcomes. In this analysis, outcomes were dichotomised into positive and negative categories, representing two groups (good versus poor), using the global item of QoL and health items as reference points (anchors). ROC curves, with 95% confidence intervals, were constructed to assess the capacity of different QOL-ACC scores to classify care recipients according to these anchor-defined groups. This approach allows the performance of a continuous scale, such as the QOL-ACC score, to be assessed for its ability to classify individuals into binary outcomes [29].

The ROC curve illustrates the discriminatory power of each possible threshold, and the area under the curve (AUC) was calculated to assess the overall classification accuracy of each QOL-ACC score with a greater area under the curve indicating higher discriminatory accuracy [30]. The AUC quantifies the probability that a randomly selected individual from the positive group has a higher QOL-ACC score than a randomly selected individual from the negative group, with values ranging from 0.5 (no discrimination, equivalent to chance) to 1.0 (perfect discrimination). Interpretation guidelines suggest that an AUC of 1.0 indicates a perfect test, 0.90–0.99 excellent, 0.80–0.89 good, 0.70–0.79 fair, 0.51–0.69 poor, and 0.5 of no diagnostic value [31–33].

For the identified optimal threshold, sensitivity (true positive rate) and specificity (true negative rate) were calculated. Positive predictive value (PPV) and negative predictive value (NPV) were also estimated to indicate the probability that individuals were correctly classified given a positive or negative result, respectively. In addition, positive and negative likelihood ratios (LR + and LR−) were calculated as prevalence-independent measures of classification performance. The LR + was calculated as sensitivity divided by (1 − specificity), and the LR − as (1 − sensitivity) divided by specificity.

There are several methods for determining the optimal cutoff value [34], each involving a trade-off between sensitivity and specificity depending on the intended application of the test [35]. In this study, for each QOL-ACC score, we selected the threshold with the highest Youden’s J statistic, which is the sum of sensitivity and specificity minus one [36]. If two adjacent thresholds had Youden’s J values differing by less than 0.05, we selected the threshold with higher sensitivity, following the methods of Giesinger et al. [37]. When the sensitivity of a threshold fell below 0.70, the closest threshold providing sensitivity near this value was chosen. All analysis were performed in Stata version 17.0 [38].

### Sensitivity analyses

Sensitivity analyses were conducted using different groupings of the global QoL and global health items as alternative anchor definitions to assess the robustness of the identified cut-off. Two sensitivity analyses were undertaken (Test 1 and Test 2). In Test 1, responses of ‘excellent’ and ‘very good’ were grouped as the positive category, while ‘good’, ‘fair’, and ‘poor’ responses were grouped as the negative category for both the global QoL and global health items. In Test 2, an unbalanced grouping approach was applied, whereby ‘excellent’, ‘very good’, and ‘good’ responses on the global health item were classified as good health, while only ‘excellent’ and ‘very good’ responses on the global QoL item were classified as good QoL. For each alternative anchor definition, the same ROC analysis was applied to the study sample, and the resulting optimal cut-off values were compared with those from the main analysis.

### Ethics statement

Ethics approval for this study was granted by the Flinders University Human Research Ethics Committee (Project No. 8399). All procedures involving human participants adhered to the ethical standards of the institutional and national research committees and complied with the 1964 Declaration of Helsinki and its later amendments. Informed consent was obtained from all participants, either online or in person, prior to data collection. All data were de-identified before analysis, and no identifiable information was retained, ensuring confidentiality and compliance with data protection requirements.

## Results

Combining both older people self-reported (*n* = 200, 62.5%) and proxy reported data (*n* = 116, 37.5%), the majority of the care recipients were female (64.2%), with mean age of 84 ± 8.2 years (range = 65–101 years). Approximately 70% of the sample was born in Australia, and mostly (94%) spoke English at home. A large proportion of care recipients had not completed Year 12 or an equivalent level of education (61.4%). Our sample was widely distributed across Australia, with 16.7% from New South Wales, 18.7% from Queensland, 8.5% from Victoria, 32.6% from South Australia, 11.1% from Western Australia, and 12.3% from Tasmania. Overall, 57.3% of respondents rated their general health as good or better on the single-item global health measure. For the global QoL item, 69.9% reported good, very good, or excellent quality of life. Half of the participants (or their proxies) scored below 17 on the QOL-ACC questionnaire (Median = 17, IQR = 14–21). The demographic characteristics of study participants were presented in Table 2.

**Table 2:** Demographic Characteristics of care recipients

Characteristics	Frequency	Percent
Data source		
Older people self-reported	200	63.3%
Proxy reported	116	36.7%
Gender		
Female	203	64.2%
Male	112	35.5%
Prefer not to define	1	0.3%
Age	Mean ± SD = 84 ± 8.2	
Born in Australia		
Yes	221	70%
No	95	30%
Speaking English at home		
Yes	297	94%
No	19	6%
Education level		
No qualifications	90	28.5%
Completed high schools	120	38%
Undergraduate degree	57	18%
Post-graduate qualification	17	5.4%
Other	32	10.1%
Residential State		
New South Wales	53	16.8%
Queensland	59	18.7%
Victoria	27	8.5%
South Australia	103	32.6%
Western Australia	35	11.1%
Tasmania	39	12.3%
General health status		
Excellent	24	7.6%
Very good	65	20.6%
Good	92	29.1%
Fair	88	27.9%
Poor	47	14.9%
Overall quality of life status		
Excellent	33	10.4%
Very good	76	24.1%
Good	112	35.4%
Fair	59	18.7%
Poor	36	11.4%
Total number of observations	316	

**Fig. 2:**
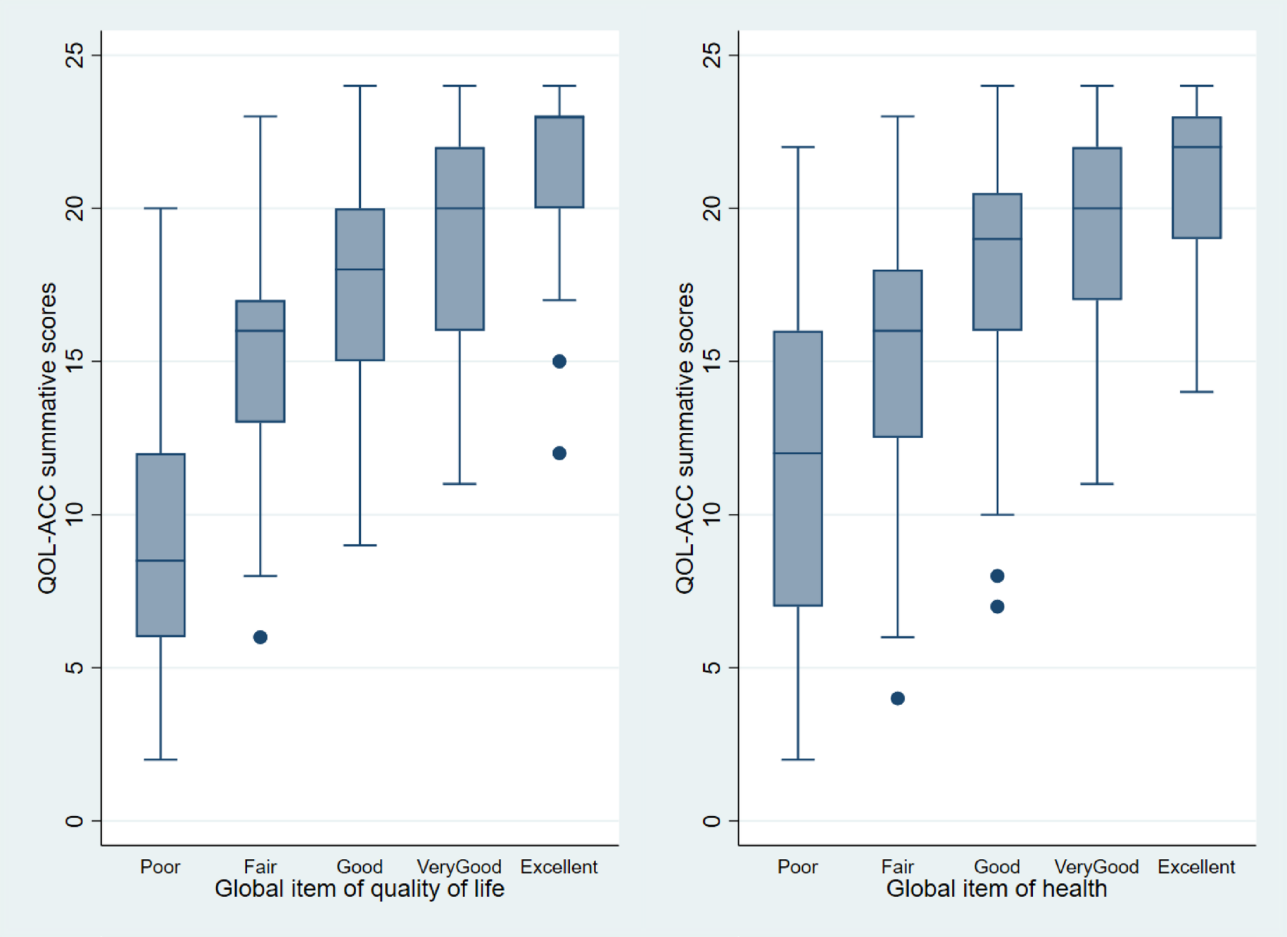
Quality of Life-Aged Care Consumers (QOL-ACC) scores by global item QoL/health categories and newly defined study groups

A positive correlation was observed between the QOL-ACC scores and the global QoL/Health categories (Fig. 2). The correlations between the QOL-ACC overall score and global item of QoL/health were 0.64 and 0.56, respectively. A potential cut-off point was observed when distinguishing between good QoL & health (G5) and poor QoL & health (G6) using global item of QoL and health as anchors.

**Fig. 3:**
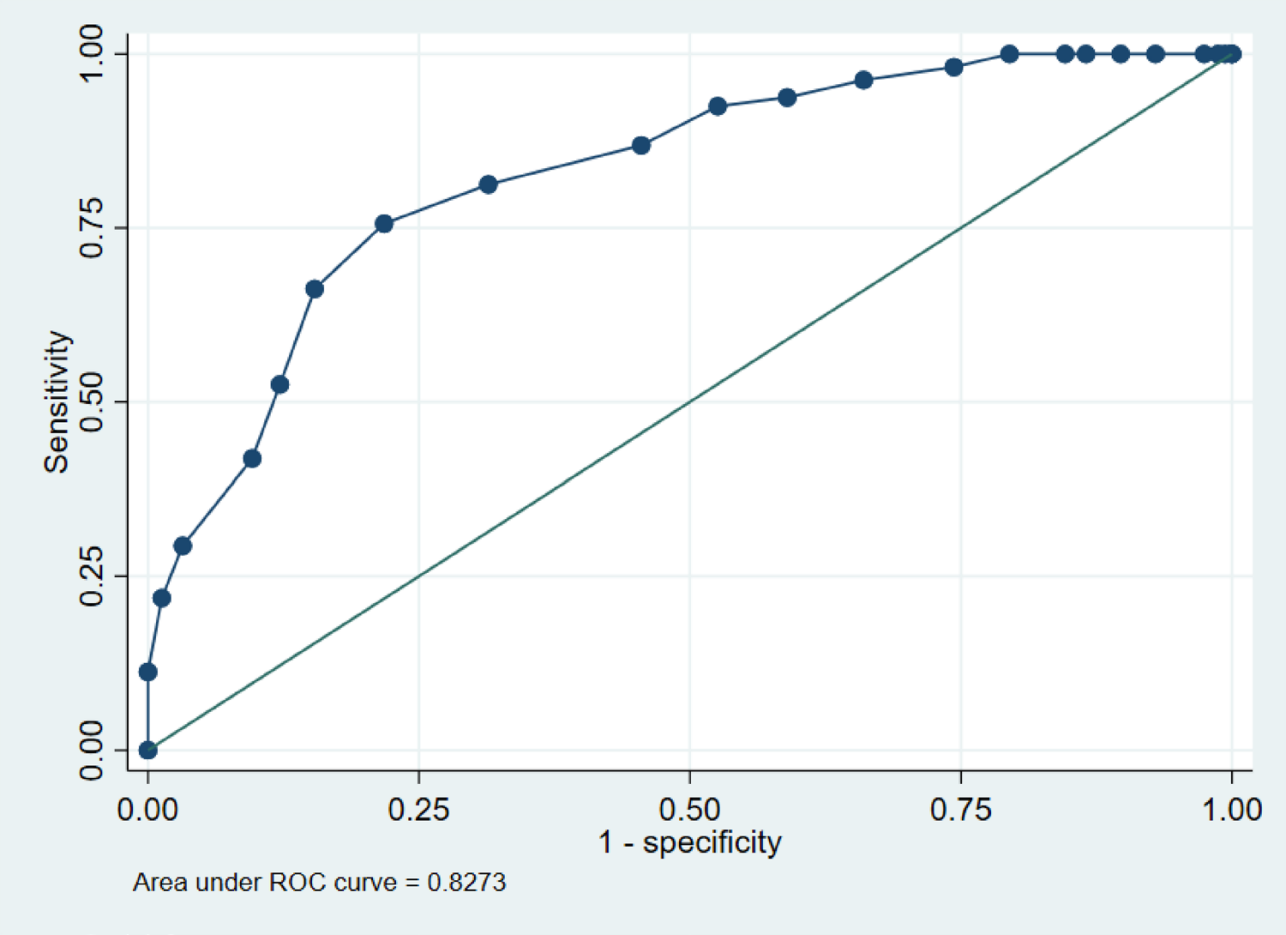
ROC curve showing sensitivity and specificity of cut-off points to predict good quality of life & health (G5) or poor quality of life & health (G6), considering the G5 and G6 groups in the sample studied

Analysis of the ROC curve identified 18 as the optimal cut-off point for evaluating ‘good’ QOL-ACC (Fig. 3). The area beneath the curve was 0.83 with 95% CI [0.78, 0.87], with a sensitivity of 76% and 78% specificity for classifying care recipients in Group G5 (good QoL & health) using a QOL-ACC cut-off of ≥ 18. With the cut-off point ≥ 18, we also got the largest Youden’s J statistic among all the QOL-ACC scores (Appendix Table 7). For Group G6 (poor QoL & health), a cut-off of < 18 yielded a sensitivity of 89% and a specificity of 61%.

Tables 3 and 4 present the classification performance of the QOL-ACC score in distinguishing care recipients classified into Group G5 and Group G6. Using a cut-off of QOL-ACC ≥ 18, the positive predictive value (PPV) was 78.06% and the negative predictive value (NPV) was 75.78%, with a corresponding positive likelihood ratio (LR+) of 3.47 and negative likelihood ratio (LR−) of 0.31 (Table 4). When applying the threshold of QOL-ACC < 18, the PPV and NPV were 40.99% and 94.84%, respectively, with LR + = 2.27 and LR− = 0.18. Sensitivity analyses indicated that the optimal cut-off was ≥ 18 in Test 1, and ≥ 19 in Test 2. A comparison of the main analysis and sensitivity analysis results is presented in Table 5.

**Table 3:** Capacity to diagnose overall quality of life, according to the cut-off points defined to screen quality of life in quality of life/health groups

Variable		G5		Total	G6		Total
		Yes	No		Yes	No	
QOL-ACC ≥ 18	Yes	121	34	155			
	No	39	122	161			
Total		160	156	316			
QOL-ACC < 18	Yes				66	95	161
	No				8	147	155
Total					74	242	316

G5: good QoL & health; G6: poor QoL & health.

**Table 4:** Indicators of capacity of diagnose QOL-ACC according to the cut-off points defined to screen quality of life in quality of life/health groups

Indicators of capacity diagnose	G5 (%) (cut-off point ≥ 18)	G6 (%) (cut-off point < 18)
Sensitivity	75.63	89.19
Specificity	78.21	60.74
Positive predictive value	78.06	40.99
Negative predictive value	75.78	94.84
Positive likelihood ratio	3.47	2.27
Negative likelihood ratio	0.31	0.18

G5: good QoL & health; G6: poor QoL & health. Positive likelihood ratio was calculated as sensitivity / (1−specificity), and negative likelihood ratio as (1−sensitivity) / specificity.

**Table 5:** Comparison of main and sensitivity analysis results

		Main analysis	Sensitivity analysis 1	Sensitivity analysis 2	
		Cut-off point: QOL-ACC ≥ 18	Cut-off point: QOL-ACC ≥ 18	Cut-off point: QOL-ACC ≥ 19	
Sensitivity		75.63%	81%	71.28%	
Specificity		78.21%	60%	70.83%	
Area under ROC curve		0.8273	0.7824	0.7953	

## Discussion

This study identified an empirically derived reference point for QOL-ACC summative scores with good discriminative ability. The threshold demonstrated balanced sensitivity and specificity and may assist interpretation of QOL-ACC outcomes in residential aged care. In the context of national implementation of QOL-ACC as a mandatory quality indicator, this reference point provides practical guidance for interpreting scores within routine aged care quality monitoring. To our knowledge, this is the first study to identify a cut-off for an aged care–specific quality-of-life assessment instrument.

The sample was predominantly female (64.2%), Australian born (70%), and English-speaking (94%), with a relatively low level of education qualification (61.4% had an educational level below Year 12 or equivalent). Compared with demographics of the general residential aged care population in Australia (60% female, 65% born in Australia, 88% speak English at home and 63.5% with highest educational qualification of below year 12) [4], the sample is broadly representative of gender, country of birth, and education attainment. The proportion speaking English at home is moderately higher in the study sample, which may slightly limit representation of culturally and linguistically diverse population.

The study sample included both self-reported and proxy-reported QOL-ACC responses. Inclusion of proxy-reported assessments reflects current practice within the Australian National Aged Care Quality Indicator Program [25], where a proportion of residents are unable to self-complete the instrument due to cognitive or functional limitations. Proxy respondents may report quality-of-life scores that differ from self-reports, and the two sources are not assumed to be interchangeable, however, excluding proxy assessments in this context would reduce the representativeness of the sample relative to the population in which the QOL-ACC is currently implemented in Australia. Previous work from our team has demonstrated that proxy-reported QOL-ACC assessments are feasible and informative for quality assessment [39]. A dedicated validation study is currently underway to further strengthen the evidence base. Accordingly, inclusion of proxy responses enhances the policy relevance and external validity of the proposed threshold, while acknowledging the inherent limitations of proxy reporting and the need for cautious interpretation [40].

For this analysis, we classified participants into two extreme groups based on the consistency of their own (or their proxy’s) ratings of the global item of QoL and health. Only respondents with aligned ratings (both good or both poor) were retained to ensure a clear distinction between high and low QoL experiences. The global QoL and health items are conceptually independent from the QOL-ACC domains but empirically related in practice [10], making them suitable as external anchor items for defining these extreme groups. In addition, as there is no fixed standard for ‘good’ or ‘poor’ QoL in aged care, the intention of this analysis is not to define a universal or absolute threshold, but to provide a population-specific reference point for interpreting QOL-ACC scores in residential aged care.

The QOL-ACC ≥ 18 cut-off demonstrated moderate sensitivity (76%) for identifying older people with good QoL among residential care recipients in Australia. Conversely, the QOL-ACC < 18 threshold showed strong sensitivity (89%) and a high negative predictive value (95%) for screening older people with poor level of QoL. While the positive likelihood ratio for detecting good QoL had minimal impact on post-test probability, the negative likelihood ratio provided meaningful information for identifying poorer QoL. This highlights the value of the cut-off as a practical flag for older people in residential care who may have low QoL and require further assessment.

Sensitivity analyses using alternative anchor groupings showed that the identified QOL-ACC cut-off was generally robust to variations in anchor specification. Although a marginally higher cut-off (≥ 19) was identified under a more restrictive anchor specification on the global QoL item (Test 2), the overall pattern of classification performance remained consistent across analyses, with only modest changes in sensitivity, specificity, and area under the ROC curve. The cut-off of ≥ 18 was retained in the main analysis and in one sensitivity test (Test 1), and it provided more balanced and stable classification performance compared with the alternative threshold. This finding is consistent with previous work by Henchoz et al. [27], who demonstrated that applying different cut-offs on the global QoL item can substantially influence empirical findings, highlighting the sensitivity of results to the choice of threshold. Overall, these results suggest that the optimal cut-off value of the QOL-ACC is influenced by anchor specification and may also vary across samples and care settings. Our aim in this study again was not to propose a universal or definitive threshold, but rather to introduce a preliminary reference point and demonstrate an exploratory, empirically grounded approach to cut-off identification that may support more practical and interpretable use of the QOL-ACC in aged care settings.

The QOL-ACC score remains a continuous measure of quality of life, and the proposed threshold is not intended to dichotomise QoL for analytical or comparative purposes. Rather, it should be viewed as a pragmatic interpretive aid designed to support applied use of the measure in real-world aged care settings. Conceptually, this use of thresholds is analogous to colour-coded score bands or risk categories commonly used in clinical and quality monitoring contexts, where thresholds act as signals to prompt further review rather than definitive classifications, consistent with guidance on the cautious use of cut-points in continuous measures [41]. Given the multidimensional and subjective nature of quality of life, any threshold should be interpreted cautiously and used alongside domain-level scores, longitudinal trends, and other indicators of resident wellbeing.

In the context of national reporting, this study’s findings align with the Quick Reference Guide for QOL-ACC under the National Aged Care Mandatory Quality Indicator Program, which defines five QOL-ACC bands: Excellent (22–24), Good (19–21), Moderate (14–18), Poor (8–13), and Very Poor (0–7) [42]. Although these categories are used for national reporting, no empirical threshold currently exists for distinguishing QoL outcomes that may warrant further attention from those that may not. Establishing an empirically derived cut-off could therefore provide a more structured framework for interpreting QoL outcomes in aged care and support monitoring, assessment of care services, and quality improvement interventions within national reporting systems.

This study adds to the literature by identifying a population-specific reference point to aid interpretation of QOL-ACC scores in residential aged care. Drawing on Australian national data, it shows how an anchor-based approach can be used to derive an interpretable threshold while accounting for context and intended use. Rather than proposing a universal standard, the results show how such reference points can assist quality monitoring and interpretation of aged care–specific QoL outcomes, including their use within quality indicator programs. This approach may also be transferable to other QoL instruments and could be explored further in future research.

This study, however, has some limitations. First, the proposed cut-off was derived using one single, modestly sized sample, and has not yet been validated in an independent cohort, which limits its generalizability. Therefore, these findings should be considered preliminary, and external validation is required before the threshold can be confidently applied in routine monitoring or policy contexts. Second, the use of a convenience sample, rather than a random or stratified sampling strategy, may limit the generalisability of the findings to the wider residential aged care population. Third, the self-report and proxy data were drawn from independent samples, and participants were recorded under separate respondent identifiers. Therefore, the likelihood of overlap is minimal, however it is theoretically possible and cannot be entirely ruled out. Finally, differences in survey administration mode (self-, interviewer-, or proxy-administered) may have introduced variability in how residents interpreted and responded to QoL items. Although this reflects routine practice within the national Quality Indicator Program and enhances real-world generalisability [43], it may also introduce mode-related response biases that should be considered when interpreting the findings.

## Conclusion

This study provides a practical, evidence-based reference point to support interpretation of QOL-ACC scores and represents the first attempt to identify a cut-off for an aged care–specific quality-of-life instrument. Use of this threshold may assist routine monitoring of quality of life in aged care, support identification of care recipients with lower level quality of life, and inform care planning, service evaluation, and quality improvement interventions. Further research is needed to examine the transferability and validity of this cutoff in home-based aged care settings and among culturally and linguistically diverse (CALD) populations, to support more equitable and high-quality aged care for older people in Australia.

## Appendix

See Tables 6 and 7.

**Table 6:** Frequency for the self-reported global item of quality of life (QoL) and health categories

Variable		Global item of QoL										Total	
		Good QoL						Poor QoL					
		Excellent		Very Good		Good		Fair		Poor			
		n	%	n	%	n	%	n	%	n	%	n	%
Global item of Health													
Good Health	Excellent	**12**	**36.4**	**6**	**7.9**	**6**	**5.4**	–	–	–	–	24	7.6
	Very Good	**12**	**36.4**	**43**	**56.6**	**9**	**8.0**	1	1.7	–	–	65	20.6
	Good	**7**	**21.2**	**18**	**23.7**	**47**	**42.0**	17	28.8	3	8.3	92	29.1
Poor Health	Fair	2	6.1	8	10.5	42	37.5	*30*	*50.9*	*6*	*16.7*	88	27.9
	Poor	–	–	1	1.3	8	7.1	*11*	*18.6*	*27*	*75.0*	47	14.9
Total		33	100	76	100	112	100	59	100	36	100	316	100

Bold area-G5: good QoL & health (n = 160); Italics area-G6: poor QoL & health (n = 74). Underline areas—Undefined (n = 82). Ns in each of those groups based on global QOL and Health ratings

**Table 7:** ROC sensitivity, specificity, and Youden’s J statistics of QOL-ACC summative scores

Cut-off point	Sensitivity	Specificity	Sum of sensitivity and specificity	Youden’s J statistic
(> = 18)	75.63%	78.21%	153.84%	53.84%
(> = 19)	66.25%	84.62%	150.87%	50.87%
(> = 17)	81.25%	68.59%	149.84%	49.84%
(> = 16)	86.88%	54.49%	141.37%	41.37%
(> = 20)	52.50%	87.82%	140.32%	40.32%
(> = 15)	92.50%	47.44%	139.94%	39.94%
(> = 14)	93.75%	41.03%	134.78%	34.78%
(> = 21)	41.88%	90.38%	132.26%	32.26%
(> = 13)	96.25%	33.97%	130.22%	30.22%
(> = 22)	29.37%	96.79%	126.16%	26.16%
(> = 12)	98.13%	25.64%	123.77%	23.77%
(> = 23)	21.88%	98.72%	120.60%	20.60%
(> = 11)	100.00%	20.51%	120.51%	20.51%
(> = 10)	100.00%	15.38%	115.38%	15.38%
(> = 9)	100.00%	13.46%	113.46%	13.46%
(> = 24)	11.25%	100.00%	111.25%	11.25%
(> = 8)	100.00%	10.26%	110.26%	10.26%
(> = 7)	100.00%	7.05%	107.05%	7.05%
(> = 6)	100.00%	2.56%	102.56%	2.56%
(> = 4)	100.00%	1.28%	101.28%	1.28%
(> = 3)	100.00%	0.64%	100.64%	0.64%
(> = 2)	100.00%	0.00%	100.00%	0.00%

Youden’s J statistic is calculated as the sum of sensitivity and specificity minus one [36]. The order of the potential cut-off point was sorted by the value of Youden’s J statistic from largest to smallest
